# The Morphology and Adhesion Mechanism of *Octopus vulgaris* Suckers

**DOI:** 10.1371/journal.pone.0065074

**Published:** 2013-06-04

**Authors:** Francesca Tramacere, Lucia Beccai, Michael Kuba, Alessandro Gozzi, Angelo Bifone, Barbara Mazzolai

**Affiliations:** 1 Center for Micro-BioRobotics, Istituto Italiano di Tecnologia, Pontedera, Italy; 2 The BioRobotics Institute, Scuola Superiore Sant’Anna, Pontedera, Italy; 3 Department of Neurobiology, Hebrew University of Jerusalem, Jerusalem, Israel; 4 Interdisciplinary Center for Neuronal Computation, Hebrew University of Jerusalem, Jerusalem, Israel; 5 Center for Neuroscience and Cognitive Systems, Istituto Italiano di Tecnologia, Rovereto, Italy; Ecole Normale Supérieure de Lyon, France

## Abstract

The octopus sucker represents a fascinating natural system performing adhesion on different terrains and substrates. Octopuses use suckers to anchor the body to the substrate or to grasp, investigate and manipulate objects, just to mention a few of their functions. Our study focuses on the morphology and adhesion mechanism of suckers in *Octopus vulgaris*. We use three different techniques (MRI, ultrasonography, and histology) and a 3D reconstruction approach to contribute knowledge on both morphology and functionality of the sucker structure in *O. vulgaris*. The results of our investigation are two-fold. First, we observe some morphological differences with respect to the octopus species previously studied (i.e., *Octopus joubini, Octopus maya, Octopus bimaculoides/bimaculatus* and *Eledone cirrosa*). In particular, in *O. vulgaris* the acetabular chamber, that is a hollow spherical cavity in other octopuses, shows an ellipsoidal cavity which roof has an important protuberance with surface roughness. Second, based on our findings, we propose a hypothesis on the sucker adhesion mechanism in *O. vulgaris*. We hypothesize that the process of continuous adhesion is achieved by sealing the orifice between acetabulum and infundibulum portions via the acetabular protuberance. We suggest this to take place while the infundibular part achieves a completely flat shape; and, by sustaining adhesion through preservation of sucker configuration. *In vivo* ultrasonographic recordings support our proposed adhesion model by showing the sucker in action. Such an underlying physical mechanism offers innovative potential cues for developing bioinspired artificial adhesion systems. Furthermore, we think that it could possibly represent a useful approach in order to investigate any potential difference in the ecology and in the performance of adhesion by different species.

## Introduction

Octopuses use their suckers to perform a remarkable variety of functions [1], such as, anchoring the body to the substrate, grasping, manipulating and investigating objects [2].

Despite several studies on this topic, spanning more than 100 years, many questions relating the function of the sucker remain unclear. Earlier studies focused on the unique features of octopus suckers, such as musculature [3], [4], [5], [6], sensing properties [7], [8], [9], [10], surface features [1], [11], grasping and coordination [1], [12], [13], as well as on function of the sucker musculature during adhesion [14], [15]. A detailed description of the anatomy of suckers on several different octopus species (namely *Octopus joubini, Octopus maya, Octopus bimaculoides/bimaculatus* and *Eledone cirrosa*) was reported by Kier and Smith [14], [15]. In these studies no particular anatomical differences were found using histology and cinematography, with the exception of a different location of muscles bundles in *Eledone cirrosa*.

Suckers are muscular-hydrostats [15], [16], [17]; their musculature, which is attached to the arms by means of extrinsic muscles (that, in turn, are covered by a continuation of the dermis and epidermis of the arms), is arranged in a three dimensional array (radial, meridional, and circular muscular fibers) that provides the skeletal-like support and the force for movement [15]. A single sucker consists of two general regions connected by a constricted orifice: the infundibulum, the exposed disk-like portion of the sucker, and the acetabulum, the upper hollow portion, which consists of a domed roof (in the upper part) and a wall region (in the remaining parts). A thin connective tissue layer, both on the internal and on the external surface, covers the entire sucker. An array of cross connective tissue fibers is gathered in the acetabular roof. The infundibulum is encircled by a rim covered with a deeply folded, loose epithelium [14], [15]. The external surface of the infundibulum is covered by the chitinous cuticle or sucker lining, which is periodically shed and continuously renewed [1], [3], [11], [18]. On the sucker surface, the cuticle bears a series of radial grooves and ridges. The ridges are organized in mammelliforme structures, composed of micrometer elementary units, called denticles.

**Figure 1:**
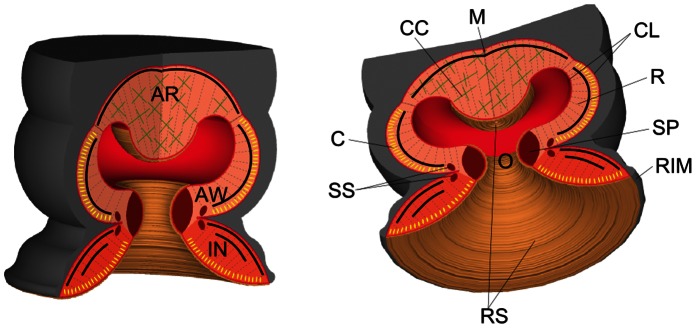
Schematic of the octopus suckers. AR, acetabular roof; AW, acetabular wall; C, circular muscle (yellow sections); CC, cross connective tissue fibers (green crosses); CL, connective tissue layer; IN, infundibulum; M, meridional muscle (black lines); O, orifice; R, radial muscle (gray dotted line); RIM, rim around the infundibulum; RS, rough surface located on the surface of the infundibulum, orifice and acetabular protuberance; SP, primary sphincter muscle; SS, secondary sphincter muscle.

Figure 1 shows a schematic view of the octopus sucker that summarizes its entire morphological structure.

The present work focuses on the study of the morphology of *Octopus vulgaris* suckers. First, we report some morphological features that are not present in other studied octopus species. Second, we use our morphological data to formulate a hypothesis on adhesion properties of *O. vulgaris* suckers. When suckers are in action, adhesion is particularly advantageous in terms of efficiency; hence we believe that a deep understanding of the undertaken natural strategy will provide important data for developing a new generation of more efficient artificial adhesion systems. The current artificial suction cups, in fact, have two main drawbacks that limit their versatility as adhesion devices. First, low efficiency (suction is limited to vacuum) and, second, a capability to only adhere to smooth surfaces [19]. These limits are completely absent in octopus suckers, which show high efficiency to perform suction (0.268 MPa below ambient pressure) [20] by means of strategic muscular arrangement and can work on almost any non-porous surfaces. We report *in vivo* ultrasonographic recordings showing the sucker in action supporting our adhesion hypothesis of *O. vulgaris* suckers.

## Materials and Methods

All animals were obtained from licensed fishermen or through the SZN (Zoological Station in Naples). All experiments were carried out in compliance with local laws. At the present no specific permits are required for the experiments performed.

The studies were conducted during years 2010 and 2011. Over that period a license was not required since European law requires a license for studies on these types of animals only from 2013. However, all facilities and procedures complied with the new EU law (Directive 2010/63/EU). Moreover, a strong effort was directed on limiting the number of animals involved in the experiments in accordance with the quality of the obtained results.

### Experimental Animals

Specimens of *Octopus vulgaris* used for histological investigation were captured from the wild, specifically in the bay of Naples, over the period March-July 2010. Histological analysis was carried out at the Stazione Zoologica Anton Dohrn, Naples, Italy. For histological experiments, animals were anesthetized by following best practice in order to minimize any induced stress. The animals were euthanized before any invasive procedure by immersion into 2l of anesthetic solution −3.5% MgCl_2_ in sea water [21]. Thirty minutes were necessary in order to reach deep anesthesia as required to euthanize the animal [22]. We used samples extracted from five different animals. The animals were dead when the suckers were explanted. The suckers were extracted from animals already designated for other studies conducted by researchers at the Stazione Zoologica Anton Dohrn of Naples. No animal was sacrificed exclusively for the extraction of octopus suckers. We were very careful to minimize the number of animals by following the 3Rs (Replacement, Reduction and Refinement) rule (Directive 86/609/EEC). For ultrasonographic investigation, specimens of *Octopus vulgaris* were captured from the wild, specifically in the bay of Livorno, over the period September-December 2010. We obtained five live animals and five dead animals from licensed fishermen. Ultrasonographic analysis was carried out at the Research Center on Sea Technologies and Marine Robotics of the Scuola Superiore Sant’Anna, Livorno, Italy. Dead animals were used for the investigation of sucker morphology; whereas, live animals were used both for the morphological investigation and *in vivo* adhesion analysis. The *in vivo* experiments were carried out on animals that were well acclimatized and used to being handled by human experimenters. All the live animals used in our investigations (500–700 g) were kept, in an enriched environment providing natural sand and rocks to allow the animals to show their full behavioral repertoire e.g. building a shelter and foraging for up to several weeks before the experiments. Temperature was controlled to be approximately 20° (+/−1°) C in a closed artificial seawater aquarium, using mechanical, physical and biological filters to provide a fast removal of organic waste products. During the experiments, they were unrestrained and free to move in a seawater filled tank (70 cm×40 cm×30 cm, 84 l) that was prepared *ad hoc* for the experimental trials. The experiment consisted in recording sucker configuration when the octopus spontaneously attached to the probe. In order to minimize the stress of the animals we did not interact with animals in a direct way and we performed experiments for a time not longer than fifteen minutes. After such time, the animals were put in their own aquarium and they did not suffer any impairment as a consequence of our experiments. Each animal was employed for only one such experiment. One week after the experiment, the animals were released in the wild.

For magnetic resonance investigation, specimens of *Octopus vulgaris* were captured from the wild, specifically in the bay of Livorno, over the period May-July 2011. We obtained three dead animals from licensed fishermen. Magnetic resonance analysis was carried out at the Center for Nanotechnology Innovation of Istituto Italiano di Tecnologia, Pisa, Italy.

### Histology

Histological analysis was carried out at the Stazione Zoologica Anton Dohrn of Naples (Italy).

Blocks of the proximal part of arm tissues including few suckers (about 2 cm total length) were removed from animals that were sacrificed by immersion in anesthetic solution.

Then, two different methods have been applied. In the first method, blocks were fixed for 48 h in 4% formalin in sea water at room temperature. Blocks were washed (in 0.1 M phosphate buffer, pH 7.6, osmolarity controlled), and crioprotected (30% sucrose in PB at 4°C for 36 h). Samples were quickly frozen (by immersion in isopentane at −80°C) and kept at the same temperature until further processing. Blocks were serially sectioned (10 µm thick) by a sliding microtome (Leica SM2010R) according to transversal and frontal planes. Transverse sections are those perpendicular to the long axis of the arm, while frontal ones are parallel to the plane defined by the opening of the sucker, as defined in [15]. Sections were stained with Milligan trichrome stain according to Kier [16].

In the second method, the tissues were fixed overnight in 4% paraformaldehyde (in 0.1 M phosphate buffer, pH 7.6, osmolarity controlled) at room temperature. They were then dehydrated in ethanol and embedded in paraffin. Blocks were sectioned by a rotary microtome (Leica RM2245); serial sections (10 µm) were obtained according to transversal and frontal planes. Sections were collected on chrome–alum–gelatin-coated slides, and stained with Picro Ponceau [16]. The sections were examined using an optical digital microscope (HIROX KH-7700).

### Ultrasonography

Ultrasonography was performed at the Centre of Marine Robotics in Livorno by means of an Esaote MyLab™One VET ultrasound imager, equipped with a 20-mm linear array (SL3116), at a frequency of 22 MHz. We performed the ultrasonographic recordings *in vivo* on animals that were unrestrained and free to move in a seawater filled tank.

We held and manually controlled the ultrasonographic probe in order to record the sucker transversal configuration when the sucker was attached to the probe. Usually, the clearer images were obtained if the sucker diameter was similar to the probe dimension.

### Magnetic Resonance Imaging (MRI)

The magnetic resonance imaging was performed on *ex vivo* suckers, using a 7 Tesla MRI scanner, and a 3D RARE spin-echo sequence with the following imaging parameters: TR = 550 ms, TE = 33 ms, RARE factor = 8, echo spacing 11 ms, and isotropic voxel size of 150 µm. The samples were scanned along their entire volume while immersed in seawater in a Plexiglas jar.

### 3D Reconstruction

A 3D model was obtained using both histological and magnetic resonance images, using a computer program for three-dimensional reconstruction (AMIRA software, Visage Imaging).

In the case of histological images, we preserved the surrounding tissues of the sucker in order to have as many natural markers as possible, for the correct alignment of the serial images. Images of serial histological sections were reconstructed with isotropic voxel size of 10×10×10 µm. The model was reconstructed by using 300 contiguous transversal histological sections, stained with Picro Ponceau, of a sucker without fiducial markers for registration.

First of all, we processed and made the sections homogeneous by using imaging filters. After completing this preliminary process, we proceeded with a manual and semi-automatic alignment of the sections.

In the case of magnetic resonance images, we used a dataset of 170 images with an isotropic voxel size of 150×150×150 µm.

At this point, in both two cases, the segmentation phase, which is the process of assigning a label to every tissue, started. As for alignment, this phase was mainly manual. Upon segmentation completion, the last step consisted in 3D surface reconstruction, an automatic process accomplished by the adopted software.

Reconstructions from histological sections (differently from magnetic resonance images) are a complex (fixation and embedding e.g. causes shrinking and possible alteration of relative sizes of different tissues) and time consuming procedure. Also, the acquisition of images, being performed manually with the aid of a microscope, does not guarantee the same reference frame for all images and therefore a manual alignment process is needed. Still, due to present constrains in the resolution of MRI, the model obtained by means of classic histological reconstruction provides a better resolution (almost 10 µm in each direction, respect to 150 µm of MRI model).

## Results

### Morphology of the *Octopus vulgaris* Suckers

**Figure 2:**
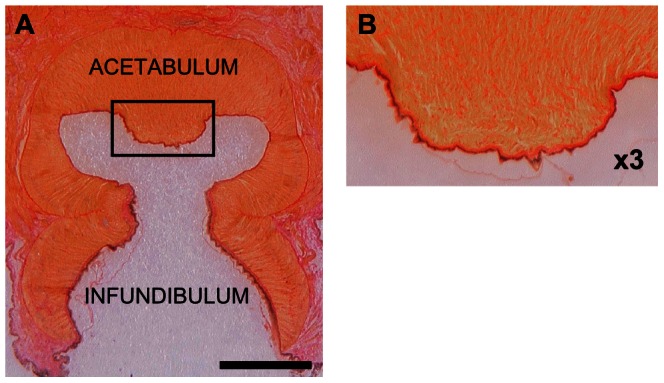
Histological transversal sections of *O. vulgaris* sucker. Picro-Ponceau staining. A) The scale bar equals 2.5 mm. In the image we can observe the protuberance at the central part of the acetabulum roof. B) Enlargement of the black box in A showing the protuberance surface roughness.

**Figure 3:**
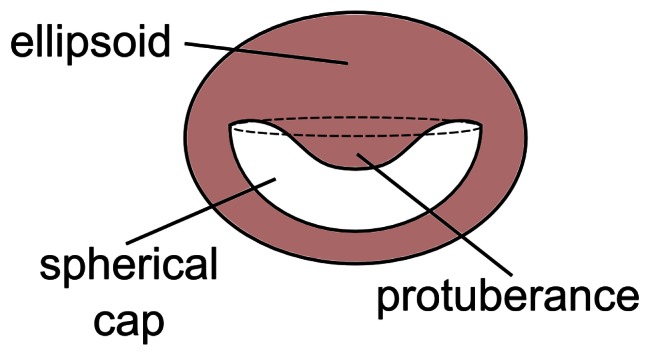
Schematic of acetabular chamber. A simplified scheme of the acetabulum where the envelope is depicted as an ellipsoid, the internal cavity as a spherical cap and the protuberance as a sort of paraboloid.

Results on the morphology of the *Octopus vulgaris* suckers, obtained by using histology, showed that the acetabulum is similar to an ellipsoid, which appeared in fact flattened at the poles (Figure 2A). The *O. vulgaris* acetabulum inside presents a spherical cap cavity with a protuberance in its central part (Figure 2A–B), as it was already reported by [3], [4] and depicted by Young [23] and Wells [24]. A simplified schematic of the acetabulum is provided in Figure 3.

**Figure 4:**
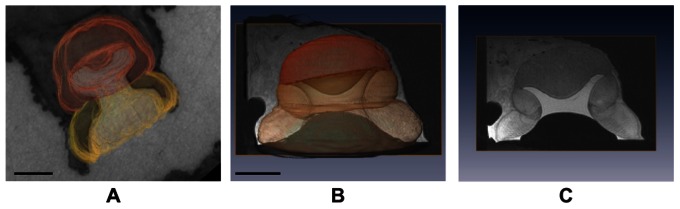
3D reconstructions of *O. vulgaris* suckers using histological and MR images. A) The scale bar equals 3 mm. B) The scale bar equals 10 mm. In both images the localization of the protuberance is evident. C) MR transversal section of octopus sucker highlighting the natural configuration of the octopus sucker structure.

This particular morphology was determined by means of MRI and 3D reconstructions of *O. vulgaris* suckers (Figure 4 and Video S1). In particular, MRI investigation clearly showed that, under a non-altered condition of the tissue, the free acetabular volume is highly negligible because the acetabular protuberance fills almost up to 80% of the entire free volume (Figure 4C). Also, we noticed that the lower part of the protuberance is very close to the orifice (few millimeters).

The CAD models obtained by means of the two 3D reconstructions (from histological and MR imaging) provide a 3D approximation of the sucker anatomical structure, as well as a useful tool to interpret and understand spatial configuration of the octopus biological samples. In fact, we used 3D reconstructions to digitally manipulate and explore, e.g. to investigate specific sub-regions, as defined by virtual sectioning planes. Figure 4 shows the result of the two 3D reconstructions obtained from the histological and MR images, which supplies spatial information on the sucker environment and structure. The presence and the localization of the protuberance in the acetabular roof are clearly evident.

**Figure 5:**
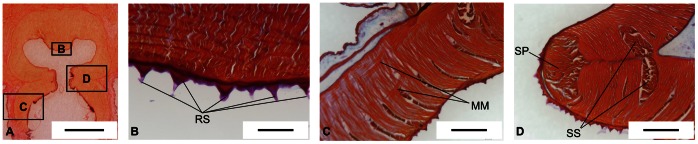
Morphological differences among *O. vulgaris* and the other octopus species. A) Transversal histological section (10 µm thick) of *O. vulgaris* sucker stained with Picro-Ponceau. B-D) Transversal histological section (10 µm thick) of *O. vulgaris* sucker stained with Milligan trichrome, showing the observed morphological differences among *O. vulgaris* and the other octopus species. B) Rough surface (RS) of acetabular protuberance. The scale bar equals 200 µm; C) Arrangement of meridional muscles (MM) in infundibular portion. The scale bar equals 600 µm; D) Primary sphincter (SP) and secondary sphincters (SS). The scale bar equals 600 µm.

Analyzing histological sections, we also noticed that the acetabular protuberance shows a thin layer of ridges (Figure 5A) - recalling the roughness that covers the internal surface of infundibulum and orifice - whereas the remaining part of the acetabulum is completely smooth.

In addition, we observed that the infundibular portion of the *O. vulgaris* sucker showed meridional muscles both in the dorsal and in the central part of the infundibulum (Figure 5B). These meridional muscles seem to originate from the same kind of fibers available in the acetabulum, passing through the acetabulum-infundibulum dividing plane and developing through the entire infundibular length.

Additionally, we confirm that, differently from the other investigated octopus species, *O. vulgaris* has three sphincters, as already figured in [25]: one primary sphincter and two secondary sphincters (Figure 5C). The primary sphincter has a cross-sectional area that is approximately 3–4 times larger than secondary sphincters (not 10 times larger, as in other described species [15]). The secondary sphincters have almost similar sizes. They are located in the lower part of the acetabulum and in the upper part of the infundibulum, respectively. They define an angle of nearly 140 degrees and they seem symmetric to the plane that separates the acetabulum from the infundibulum (Figure 5B).

### Ultrasonographic Recordings of Adhesion Mechanism

The morphological configuration of *Octopus vulgaris* suckers and their adhesion capabilities were investigated and validated by means of ultrasonographic imaging during the adhesion process.

**Figure 6:**
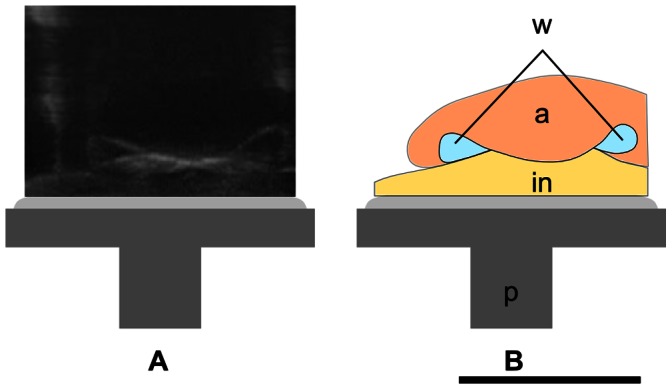
*Octopus vulgaris* sucker during adhesion process. A) Ultrasonography of middle transverse section of sucker attached to the ultrasonographic probe. B) Schematic of A: a, acetabulum; in, infundibulum; w, water; p, ultrasonographic probe. The acetabular protuberance is in contact with the upper part of the side walls of the orifice. The scale bar equals 1 cm.

We observed that when suckers come in contact with the substrate the infundibular part changes its shape, from a conical shape to a completely flat shape, whereas the acetabular part remains almost unchanged. It is worth noting that during the adhesion configuration, the acetabular roof protuberance is in contact with the upper part of the side walls of the orifice (Figure 6 and Video S2). This way, the orifice closure forms two water compartments in the sucker corresponding to: the torus of water that is created in the acetabular cavity around the protuberance itself; and, the volume of water created between the lower part of the acetabular protuberance and the adhesion substrate.

Furthermore, a major remarkable aspect of the sucker behavior emerges when trying to detach the sucker from an external object: despite the force applied to separate the object, the acetabulum shape remains the same and, specifically, with the protuberance being always in contact with the upper part of the side walls of the orifice.

## Discussion

### Morphology of the *Octopus vulgaris* Suckers

In this study we show that *O. vulgaris* has an evident protuberance on the acetabular roof that protrudes toward the orifice. This protuberance is characterized by a rough surface whereas all the remaining part of the acetabulum is completely smooth. This anatomical structure has not been found in the acetabulum of other studied octopus species, which is instead described in the literature as a hollow spherical cup without any protrusions and with a completely smooth surface. Our investigation on *O. vulgaris* suckers also highlights a characteristic number of muscles of the sphincters and the distribution of meridional muscles in the infundibular portion.

The present study uses different methods of morphological observation. To the best of our knowledge, this represents the first example of MRI analysis, in octopus investigation. Together with *in vivo* ultrasonography, these methods represent novel powerful tools to confirm and expand standard histological approaches. These data were used to formulate a novel hypothesis on the mechanism of maintaining suction over extended periods of time. It is worth remarking that the information obtained from all three techniques converge to describe a consistent sucker morphological structure.

### Hypothesis of *Octopus vulgaris* Suckers Adhesion Mechanism

The novel identified anatomical features led us to investigate the role of the acetabular protuberance in the sucker adhesion and detachment processes. Previous studies on *Octopus vulgaris* sketched the acetabular protuberance [3], [4] and supplied a gross anatomical description. In addition, Girod [3] presented a model of the *O. vulgaris* sucker adhesion mechanism; nevertheless, this model was considered imprecise and contradictory [4], [15] and it did not assign a specific role to the protuberance. Kier and Smith studied the morphological structure of different octopus species (*Octopus joubini, Octopus maya, Octopus bimaculoides/bimaculatus, and Eledone cirrosa*), and proposed their model on the sucker adhesion mechanism based on their findings in these species [14], [15].

**Figure 7:**
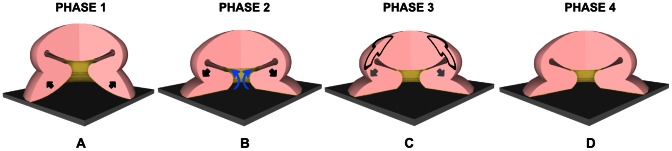
Schematic view, in four phases, of the adhesion mechanism proposed for the *O. *vulgaris sucker. A) Forming of a tight seal that prevents water from leaking at the rim. Infundibular radial muscles begin to contract (black arrows) to increase the contact area between (flattened) infundibulum and substrate; B) Contraction (black arrow) of acetabular radial muscles creates suction and moves water from infundibulum-substrate interface toward the acetabulum (blue arrows), as well, enhancing attachment; C) Meridional muscles of acetabulum contract (black arrows), allowing the protuberance makes contact with the upper part of the side walls of the orifice; meanwhile, the acetabular radial muscles are still contracted (gray arrow). Rough surfaces of both orifice and acetabular roof (coming into contact) contribute to adhesion. A torus of water is created in the acetabular cavity around the protuberance itself; D) Acetabular radial and meridional muscles stop to contract. The protuberance is passively kept in contact with the upper part of the side walls of the orifice, due to the cohesive force of water in the infundibular compartment and the friction of the two roughness surfaces that are in contact (acetabular protuberance and upper part of side walls of orifice). These two forces are balanced by the elastic restoring force of acetabular protuberance.

According to our morphological investigation and ultrasonographic recording results, we hypothesize that *O. vulgaris* sucker adheres by sealing and suction (as already described in literature) (Figure 7A and B); sealing the orifice between acetabulum and infundibulum portions via the acetabular protuberance, with the infundibular part achieving a completely flat shape (Figure 7C); sustaining adhesion through preservation of sucker configuration (Figure 7D).

As claimed in Kier and Smith’s model [14], [15], in the first phase of the adhesion process, the infundibulum, being very dexterous and flexible, can arrange its shape depending on the surface, so as to define a seal that prevents water from leaking. Due to the constant volume of the muscular hydrostat system and in order to maximize the adhesion force, the contraction of the infundibular radial muscles reduces the thickness of the infundibulum, while increasing the adhesion surface (Figure 7A). At this first stage, although the internal water volume in the sucker is isolated from the external one, there is no pressure difference.

Once the seal is formed, as described by Kier and Smith [14], [15], the contraction of acetabular radial muscles puts the water inside the sucker in tension (Figure 7B). Such water volume has a high bulk modulus; therefore, it resists to expansion and the tension force is balanced by pressure reduction. We argue that this phenomenon mainly permits negligible residual water (at the infundibulum-substrate interface) to move towards the acetabular chamber, by flowing through the dense network of grooves available on the attached infundibulum portion. As a consequence, the water at infundibulum-substrate interface is minimized and sucker adhesion takes place. In addition, the ultrasonographic recordings show that during adhesion the acetabular protuberance is in contact with the upper part of the side walls of the orifice locking it up, and a torus of water is created in the acetabular cavity around the protuberance itself (Figure 7C). Meridional muscles might perform the orifice closure, since they extend from the apex of the acetabular roof toward the bottom of the sucker (i.e. infundibulum), their contraction should cause little crushing at the poles, as needed for protuberance-orifice contact. At this stage, two isolated water compartments in the sucker are created, separated by means of the orifice closure: the first (i.e. torus of water) is located in the acetabular chamber and the second in the infundibular portion between the orifice and the substrate. We suppose that, when the locking up of the orifice occurs, the acetabular radial muscles are still contracted and the pressures remain equal in the two compartments and still lower than the external pressure.

**Figure 8:**
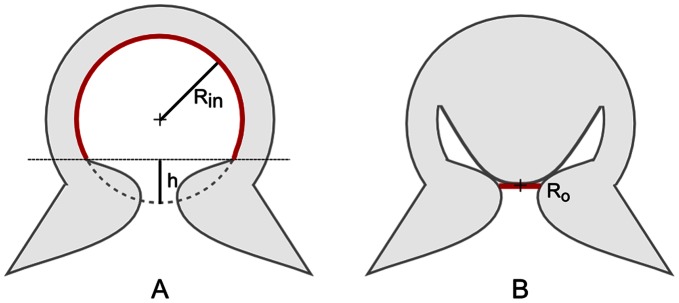
Schematic of the surfaces of action involved in adhesion. A) In Kier and Smith model [14], [15] the acetabular chamber is depicted as a hollow spherical cavity. R_in_ is the acetabular internal radius and h is the height of spherical cap that does not take part to suction. B) In the model proposed in this work the acetabular chamber presents a protuberance acting on the orifice and maintaining low pressure in the infundibular portion of the sucker. R_o_ is the radius of the orifice.

At the last stage of the adhesion process (Figure 7D), we suppose that the acetabular radial muscles stop contracting. We can thus argue that pressure in the acetabular compartment increases, but pressure in the infundibular compartment remains unchanged, since it is isolated from the acetabulum by means of orifice closure. In the light of this, the protuberance maintains contact with the upper part of the sidewalls of the orifice due to the cohesive force of water volume in the infundibular compartment. At this stage, no muscles are contracted. The passive elastic force of the acetabular tissues, which tend to return in rest configuration, counterbalances the above cohesive force. This phenomenon maintains suction at the water interface (Figure 7D). Thus, in our hypothesis the acetabular radial muscles are active at the beginning of the adhesion process, in order to remove the water from infundibular compartment and to initiate the contact between the acetabular protuberance and the upper part of the orifice sidewalls. Therefore, in our model the passive elastic restoring force of the acetabular protuberance performs sucker attachment over extended periods of time. Further support to our model is given by estimating the forces involved in the adhesion process. If we consider the acetabulum as a hollow spherical structure, the surface of action of the muscles responsible for generating the suction (acetabular radial muscles) is equal to 3πR^2^_in_, which results from 4πR^2^_in_–2πR_in_h (where R_in_ is the acetabular internal radius and h is the height of the spherical cap that does not take part in suction) and by approximating h to ½R_in_, as shown in Figure 8A. Instead, in case of the observed *O. vulgaris* sucker morphology, the surface of action of the main element responsible for generating suction (acetabular protuberance) is the orifice opening, which is equal to πR_o_^2^, where R_o_ is the orifice radius (Figure 8B). Considering that the pressure is by definition a force per unit of area, if the surface of action decreases, the force needed to achieve the same suction pressure decreases as well. For example, by approximating R_o_ to ½R_in_, in our model the force needed to generate suction would be reduced to 1/12 the force needed in case of hollow spherical acetabulum.

Also, it is worth noting that our hypothesis suits the distribution of roughness on the surface of the protuberance and on the upper part of the sidewalls of the orifice. Such a feature adds friction and thus gives a greater stability to the contact between the two parts (acetabular protuberance and upper part of the side walls of the orifice).

This theory seems to be sustained by studies carried out by Varenberg and Gorb [26], who demonstrated that fibrillar microstructures are preferred to flat surfaces in applications where a total attachment force must be generated in a binary on/off state (such as in the case of octopus suckers). Also, Varenberg and Gorb [27] reported that structured surfaces, as for instance the infundibulum and acetabular protuberance surfaces in the *O. vulgaris* sucker, reveal a 25% increase in pull-off force when immersed in water, and their underwater attachment is 20 times more effective than that of flat surfaces. These studies indicate that roughness could be considered as a typical feature of surfaces performing wet adhesions (like, for example, the infundibular portion).

As regards detachment of the sucker from the substrate, we think that the circular muscles of the infundibulum contract in order to release the seal. At the same time, the contraction of the acetabular circular muscles increases the pressure in the acetabulum, which, by pushing the torus of water toward the orifice, induces detachment between acetabular protuberance and the upper part of the sidewalls of the orifice.

As a result, in our adhesion model, radial and meridional muscles are responsible for the adhesion phase, whereas the circular muscles are responsible for the detachment phase.

There is evidence in support of this hypothesis from ultrasonographic recordings, where the contact between protuberance and opening during adhesion is visible (Figure 6 and Video S2).

Besides proposing a novel adhesion mechanism for the *O. vulgaris* suckers, the present study offers potential cues for the development of innovative, bioinspired artificial adhesion strategies and devices able to outperform the currently available solutions. Furthermore, we think that this work presents a useful approach towards investigating potential differences in ecology and performance of sucker adhesion by different species.

## Supporting Information

Video S1 **3D Reconstructions of** ***Octopus vulgaris*** **suckers from histological sections and magnetic resonance images.**
(AVI)

Video S2 ***In vivo*** **ultrasonographic recording of the adhesion process performed by** ***Octopus vulgaris*** **.**
(MP4)
